# Supplementary figures and images for: Whole genome sequencing of Streptococcus pneumoniae: development, evaluation and verification of targets for serogroup and serotype prediction using an automated pipeline
Source: PeerJ. 2016 Sep 14;4:e2477. doi: 10.7717/peerj.2477 (PMC5028725; doi:10.7717/peerj.2477)

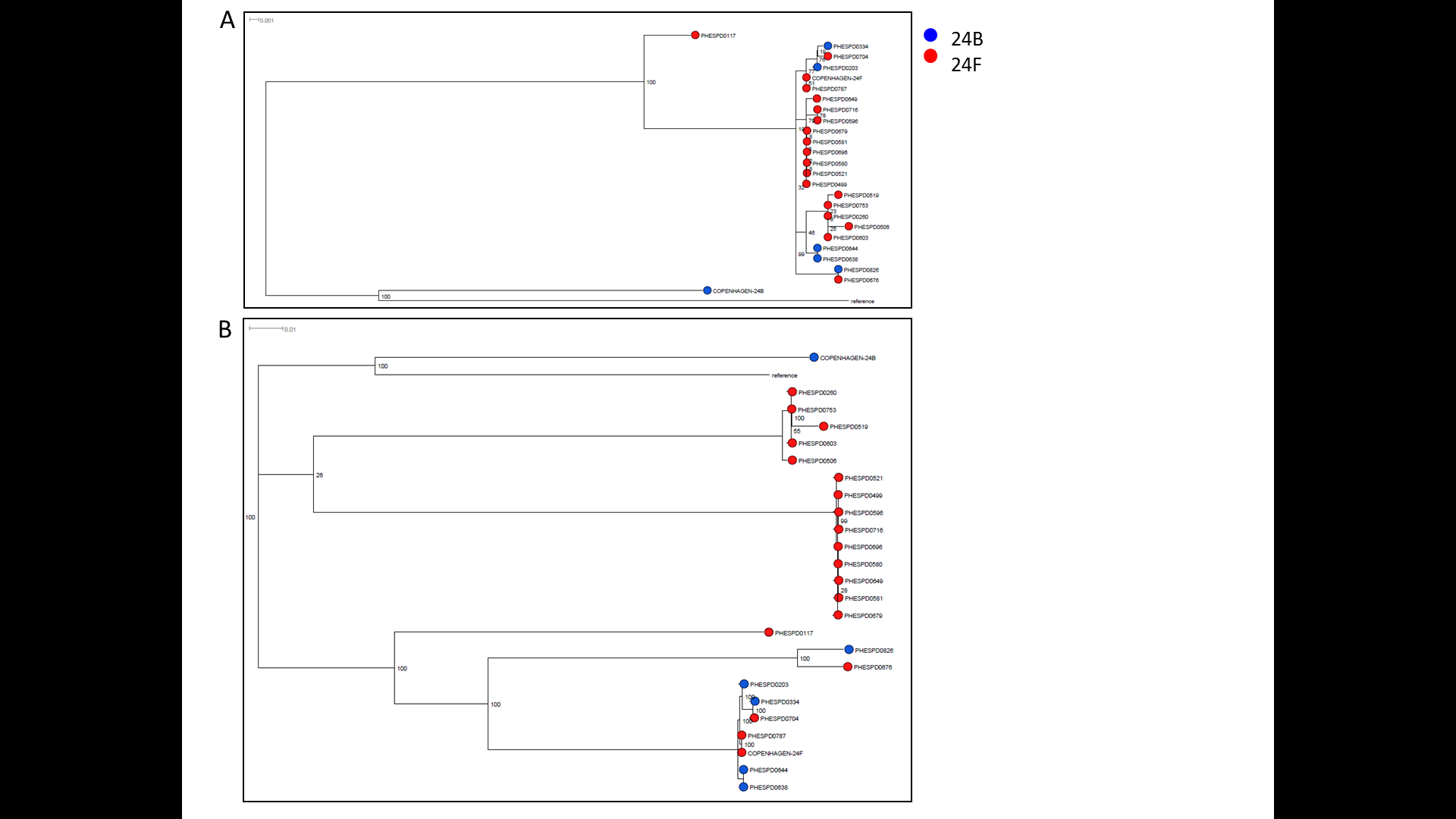

Supplement: Figure S1 — Maximum likelihood trees following SNP analyses using (A) capsular locus sequence from Streptococcus pneumoniae strain 24F L ( CR931688); and (B) complete genome of non-capsular Streptococcus pneumoniae R6 strain. The percentage of replicate trees in which the associated taxa clustered together in the bootstrap test (1000 replicates) are shown next to the branches. The scale bar corresponds to the number of nucleotide substitutions per site. The analysis involved 26 nucleotide sequences. All positions with less than 90% site coverage were eliminated. That is, fewer than 10% alignment gaps, missing data, and ambiguous bases were allowed at any position. There were a total of (A) 145 and (B) 19,123 positions in the final dataset. Evolutionary analyses were conducted using RAxML. [file peerj-04-2477-s001.png]
